# CREATIVITY, HOPE, AND EXPECTATION IN A POETRY PROGRAM: RETHINKING WHAT COUNTS AS SUCCESS IN DEMENTIA CARE

**DOI:** 10.1093/geroni/igz038.1557

**Published:** 2019-11-08

**Authors:** Kate de Medeiros

**Affiliations:** Miami University, Oxford, Ohio, United States

## Abstract

Creativity offers liberation from the framework of decline in later life in general, and within the context of dementia specifically. Cohen described the synergy of hope and expectation as ways through which people access their creative potential, especially in the face of loss. This paper explores three case drawn from a qualitative study. Eight residents living in a secure dementia care facility participated in six 30-minute interactive poetry sessions using guidelines from the Alzheimer’s Poetry Project (APP). Observations were conducted at baseline, during the intervention, and one week afterwards. All sessions were audio recorded, transcribed and analysed. Important findings included the positive potential of imagined futures, creative engagement as a tool for building social bonds, and the importance of language play. Overall, these findings point to new ways to consider success in dementia interventions by focusing on the potential to create meaningful and creative engagement opportunities.

